# Amyloid Properties of the Mouse Egg Zona Pellucida

**DOI:** 10.1371/journal.pone.0129907

**Published:** 2015-06-04

**Authors:** Nathan Egge, Archana Muthusubramanian, Gail A. Cornwall

**Affiliations:** Department of Cell Biology and Biochemistry, Texas Tech University Health Sciences Center, Lubbock, Texas, United States of America; University of Maryland School of Medicine, UNITED STATES

## Abstract

The zona pellucida (ZP) surrounding the oocyte is an extracellular fibrillar matrix that plays critical roles during fertilization including species-specific gamete recognition and protection from polyspermy. The mouse ZP is composed of three proteins, ZP1, ZP2, and ZP3, all of which have a ZP polymerization domain that directs protein fibril formation and assembly into the three-dimensional ZP matrix. Egg coats surrounding oocytes in nonmammalian vertebrates and in invertebrates are also fibrillar matrices and are composed of ZP domain-containing proteins suggesting the basic structure and function of the ZP/egg coat is highly conserved. However, sequence similarity between ZP domains is low across species and thus the mechanism for the conservation of ZP/egg coat structure and its function is not known. Using approaches classically used to identify amyloid including conformation-dependent antibodies and dyes, X-ray diffraction, and negative stain electron microscopy, our studies suggest the mouse ZP is a functional amyloid. Amyloids are cross-β sheet fibrillar structures that, while typically associated with neurodegenerative and prion diseases in mammals, can also carry out functional roles in normal cells without resulting pathology. An analysis of the ZP domain from mouse ZP3 and ZP3 homologs from five additional taxa using the algorithm AmylPred 2 to identify amyloidogenic sites, revealed in all taxa a remarkable conservation of regions that were predicted to form amyloid. This included a conserved amyloidogenic region that localized to a stretch of hydrophobic amino acids previously shown in mouse ZP3 to be essential for fibril assembly. Similarly, a domain in the yeast protein α-agglutinin/Sag 1p, that possesses ZP domain-like features and which is essential for mating, also had sites that were predicted to be amyloidogenic including a hydrophobic stretch that appeared analogous to the critical site in mouse ZP3. Together, these studies suggest that amyloidogenesis may be a conserved mechanism for ZP structure and function across billions of years of evolution.

## Introduction

The zona pellucida (ZP) is an extracellular matrix surrounding the mammalian oocyte that carries out multiple functions during fertilization including protection from polyspermy and cross-species fertilization. The formation and structure of the mouse ZP in particular has been extensively studied. The mouse ZP is formed exclusively by three glycoproteins, ZP1, ZP2, and ZP3 [1]. Each ZP protein is produced by the oocyte and is shuttled to the cell membrane where it is anchored by a transmembrane domain and cleaved by a furin-like protease to yield the mature extracellular form [2, 3]. As the ZP forms around the growing oocyte, the ZP proteins polymerize into filaments forming the three-dimensional ZP matrix [4, 5]. All ZP proteins contain a conserved ZP polymerization domain that is further divided into N-terminal (ZP-N) and C-terminal (ZP-C) subdomains. Although each ZP protein can form homopolymers, formation of ZP filaments requires interaction between ZP3 (type I ZP domain subunit) and ZP1 or ZP2 (type II ZP domain subunit) proteins [3, 6]. In addition to the ZP polymerization domain present in all ZP proteins, ZP1 and ZP2 also possess additional ZP-N repeats in N-terminal extensions of the protein. These ZP-N repeat domains are found only in ZP/egg coat proteins in species whose ZP is responsible for species-specific interactions with spermatozoa suggesting an important role for the ZP-N repeat domain in gamete recognition [7].

X-ray crystallographic studies of the ZP-N subdomain of mouse ZP3 showed it adopted a unique IgG-like fold with eight β-strands forming an antiparallel β-sandwich [7]. Subsequent structural analysis of the ZP polymerization domain (ZP-N + ZP-C) of chicken ZP3 revealed that the ZP-C subdomain also adopts a β-sandwich fold with the same topology as ZP-N suggesting both subdomains may have evolved from a single ancestral IgG-like domain [8]. The ZP domain crystallized as a domain swapped dimer arranged in antiparallel orientation with the two ZP molecules held together by interactions between ZP-N and ZP-C subdomains from opposite subunits [8]. Despite these elegant studies, however, little is known regarding the mechanism by which ZP fibrils form and how this structure participates in gamete recognition.

Proteins that are homologous to mammalian ZP proteins have been found in the egg coat surrounding oocytes in non-mammalian vertebrates as well as in invertebrates suggesting that the basic structure and function of the egg coat/ZP is conserved [9]. Indeed, egg coat/vitelline envelope proteins possess ZP polymerization domains and the ability to form fibrils that create three-dimensional matrices [9]. The presence of a ZP-N-like domain in the yeast mating protein α-agglutinin/Sag 1p, and its necessity for binding to haploid cells of opposite mating type, suggests the basic mechanism of gamete recognition and parallel processes in yeast may be evolutionarily related [10, 11]. However, sequence similarity between ZP polymerization domains across species is low and the mechanism that is responsible for the commonality in ZP structure and its role in gamete recognition has not been determined.

Studies in fish and in the silk moth have suggested that the egg coats (chorion) surrounding the oocytes are functional amyloid structures [12–14]. More recently, peptide analogues of the ZP-N domain from human ZP1 were shown to form amyloid in vitro [15]. Based on these previous studies and that the mouse ZP also is a filamentous matrix, prompted us to examine whether it is an amyloid. Amyloids are proteins that self-aggregate and form highly ordered cross β-sheet fibrillar structures. Functional amyloids, defined as amyloids that carry out biological roles in the absence of pathology, have long been known to exist in bacteria and yeast [16, 17]. Although in mammals amyloids typically have been associated with neurodegenerative diseases including Alzheimer, Parkinson, and Lou Gehrig’s disease, and prion diseases such as bovine spongiform encephalopathy, accumulating evidence suggest that they can also perform functional roles. For example, the Pmel protein forms amyloid in melanosomes where it functions as a stable scaffold for the synthesis of melanin, while in the pituitary gland several hormones are stored as amyloids in the secretory granules until their release into monomers following secretion [18, 19]. Amyloid structures are also involved in programmed necrosis (necroptosis) in which the RIP1 and RIP3 proteins interact to form an amyloid core in the necrosome [20].

Functional amyloids are also present within the reproductive tract. We have previously shown that functional amyloids are present in the mouse epididymal lumen and in the sperm acrosomal matrix suggesting roles in sperm maturation and in sperm-ZP interactions, respectively [21, 22]. Herein, we present evidence suggesting that the mouse ZP is also a functional amyloid. Furthermore, using an algorithm to predict amyloidogenic sites, we show that the ZP polymerization domains of egg coat homologs of ZP3 from five different taxa, including marine invertebrates, as well as the ZP-N domain in the yeast mating protein α-agglutinin/Sag 1p, possess amyloidogenic regions comparable to that in the mouse ZP3 protein. Similarly, amyloidogenic sites in the ZP-N repeat domains of mouse ZP1 and ZP2 are also present in the ZP-N repeat 10 in the abalone vitelline envelope protein VERL. Based on the crystal structures of the ZP-N and ZP polymerization domains of ZP3, most of the predicted amyloidogenic sites localized to amino acids that form β-strands, several of which are predicted to be important sites for protein-protein interactions. Together, these studies suggest that amyloidogenesis may be a conserved mechanism for ZP structure and its functional role in gamete recognition.

## Materials and Methods

### Animals/ Superovulation

3-week old female C57BL/6 mice were purchased from Charles River Laboratories (Wilmington, MA). Mice were housed under a 12 h light: 12 h dark cycle with free access to food and water. Mice (3 to 5-weeks old) were induced to superovulate by i.p. injection with 8 IU pregnant mare serum gonadotropin (cat. no. G4877, Sigma Chemical Co., St. Louis, MO) at 5:00pm, followed 48 hours later with i.p. injection of 8 IU human chorionic gonadotropin (hCG, cat. no. C1063, Sigma Chemical Co., St. Louis, MO). Oviducts were harvested 15–16 hours after hCG injection. All animal studies were conducted in accordance with the principles and procedures outlined in the National Institutes of Health Guidelines for Care and Use of Experimental Animals. This work was approved by the Texas Tech University Health Sciences Center IACUC under protocol 94041.

### Isolation of Oocytes and ZP

All steps were performed in embryological watch glasses using a dissecting microscope. One oviduct at a time was placed in sterile PBS (137 mM NaCl, 2.68 mM KCl, 8.1 mM Na2HPO4, 1.47 mM KH2PO4, pH 7.4) supplemented with 100 IU/ml penicillin and 100 μg/ml streptomycin. Oviducts were punctured with a 30 gauge needle to release cumulus-oocyte complexes. Complexes were then transferred using a pulled glass Pasteur pipette to PBS containing 0.3 mg/ml hyaluronidase (cat. no. H3884, Sigma Chemical Co., St. Louis, MO) and incubated for 2 min at 37°C to allow cumulus cells to disperse. Cumulus-free oocytes were then transferred with pulled glass pipettes through two rinses of fresh PBS. ZP were isolated from oocytes by repeated pipetting of multiple oocytes through a glass pulled Pasteur pipette with inner diameter ~25 μm [1]. ZP were then washed through three changes of PBS to remove any remaining cellular debris. In some experiments, intact oocytes were incubated in 90% DMSO (wt/wt) for 2 minutes at room temperature and the dissolution of the ZP monitored visually with a dissecting microscope.

### Immunofluorescence Analysis

All steps for ZP immunofluorescence were performed in embryological watch glasses in 500 μl volumes. For OC and A11 immunostaining, isolated ZP were blocked in 50% goat serum (GS) (Invitrogen/ThermoScientific, Waltham, MA) in PBS for 1 h at RT. ZP were transferred to PBS-1% BSA (cat. no. A7511, Sigma Chemical Co., St. Louis, MO) containing either rabbit anti-amyloid fibrils OC (1:1000, cat. no. AB2286, EMD Millipore, Bedford, MA) or rabbit anti-amyloid oligomers A11 (1:1000 cat. no. AB9234, EMD Millipore) antibodies and incubated for 2 h at RT. Normal rabbit serum (NRS) at 1:1000 was used as control. ZP were washed 3 x with PBS and blocked as done previously and then incubated 30 min at RT in 2 μg/ml goat anti-rabbit conjugated Alexa Fluor 488 secondary antibody (cat. no. A11006, Invitrogen/ThermoScientific Waltham, MA) diluted in PBS-1% BSA. ZP were then washed 3 x in PBS, transferred to 60 μl PBS drops on slides, and mounted with coverslips with paraffin wax applied to each corner to prevent breakage of the oocytes.

For ZP3 immunostaining, isolated ZP were blocked in 10% chicken serum (CS) (Vector Laboratories, Burlingame, CA) in PBS for 30 min at RT, then incubated for 2h at RT in 2 μg/ml goat anti-human ZP3 IgG (N-20, cat. no. sc-23715, Santa Cruz Biotechnology, Santa Cruz, CA) in PBS-1% BSA. Normal goat IgG (2 μg/ml) was used as a control antibody. ZP were washed 3 x in PBS, blocked as before, and then incubated in 0.2 μg/ml chicken anti-goat conjugated Alexa Fluor 594 (cat. no. A21468, Invitrogen/ThermoScientific, Waltham, MA) diluted in PBS-1% BSA. ZP were washed 3 x in PBS and mounted on to slides in 60 μl PBS drops. All samples were examined immediately following mounting on to slides. Images were captured using a Zeiss Axiovert 200 microscope equipped with epifluorescence using filters with excitation at 545 to 580 nm and emission > 610 nm.

### Thioflavin S and Congo Red Staining

Thioflavin S (ThS) (Sigma Chemical Co., St. Louis, MO) was prepared as a 1% stock solution in PBS, filtered and stored in the dark. Isolated ZPs were transferred to 0.1% ThS diluted in PBS and incubated for 2 h at RT in the dark. ZPs were then washed 3 x in PBS and mounted onto slides in 60 μl PBS drops and cover slipped. Congo Red was prepared as a 0.2% solution in 427 mM NaCl, filtered and stored in the dark. Isolated ZP were incubated overnight in 0.2% Congo Red in a watch glass at 4°C. ZP were then pelleted by centrifugation at 15000 x g for 5 min and the pellet resuspended in 0.25% SDS for 2 min to partially disperse the ZP. The ZP were centrifuged again and the pellet resuspended in PBS and allowed to dry on a slide. After washing with water to remove any remaining SDS, the ZP pellet was stained with 0.2% Congo Red for 2 hr at room temperature, washed several times with water, and then cover slips mounted using Fluoromount G. ThS and Congo Red stained ZP were examined with a Zeiss Axiovert 200 M microscope using filters with excitation at 425 nm and emission at >475 nm for ThS and excitation at 545–580, emission > 630 nm for Congo Red and images captured using a coupled device camera (CCD; AxioCam MRc, Zeiss) and Axiovision software version 4.5. Congo Red stained ZP were also imaged with differential interference contrast (DIC) to detect birefringence.

### Dot Blot Analysis

Isolated ZP (35 each) were transferred in a small volume (about 1–2 μl) to a 500 μl microfuge tube containing 10 μl of 90% DMSO (wt/wt) (tissue culture grade, Sigma Chemical Co., St. Louis, MO), 1% SDS, or 0.25% SDS. ZP in DMSO were incubated for 90 min at RT, whereas ZP in 1% or 0.25% SDS were incubated at RT for 10 and 4 min, respectively. PBS (184 μl) and 6 μl 100% methanol were added to each tube, and enough SDS was added to bring all samples to 0.05% SDS. Samples were blotted onto 0.1 μm nitrocellulose membrane (Protran BA79, cat. no.10402062, Whatman, Dassel, Germany) using a Dot Blot 96 vacuum apparatus (cat. no. 053–401, Biometra, Göttingen, Germany). Membranes were first equilibrated in TBS (50 mM Tris-HCl, 200 mM NaCl, pH 7.4) for 5 min at RT, and then placed in the dot blot apparatus. Each well in use was pre-wet with 100 μl TBS and vacuumed dry at 2–3 mmHg. 200 μl of sample was then loaded into each well and vacuumed dry. Wells were then rinsed with 200 μl TBS containing 0.2% Tween-20 (TBST) and vacuumed dry. Some wells received buffer alone in place of sample. Blots were blocked in 3% nonfat dry milk in TBST with shaking for 1 h at RT, and then incubated overnight at 4°C with either 1:10,000 rabbit anti-amyloid fibrils OC or 1:10,000 rabbit anti-amyloid oligomer A11 antibodies in 3% nonfat dry milk in TBST. Blots were then washed 3 x 10 min in TBST with shaking, and incubated with 1:20,000 goat anti-rabbit conjugated horseradish peroxidase (HRP) secondary antibody (cat. no. 65–6120, Invitrogen/ThermoScientific, Waltham, MA) for 2 h at RT. The blots were washed 6 x 5 min in TBST and the bound enzyme was detected by chemiluminescence (Supersignal West Femto, cat.no.34095, ThermoScientific, Waltham, MA) following the manufacturer’s directions.

### Protein Aggregation Disease (PAD) Pulldown

Thirty ZP were transferred to a microfuge tube containing 200 μl PBS and amyloids isolated using the protein aggregation disease (PAD) kit (PADB100, Microsens Biotechnologies, London, UK) following the manufacturer’s instructions. Proteins were eluted off the PAD beads by incubation in 1x reducing SDS-PAGE loading buffer containing 2% SDS, 4% β-mercaptoethanol at 70°C for 15 min. Beads were captured by magnet and supernatant (eluent) saved for Western blot analysis. In some experiments, 30 ZP were incubated in 90% DMSO in PBS for 90 min at RT prior to PAD pulldown. PBS buffer alone was also incubated with the PAD beads.

### Immunoblot

PAD pulldown samples were resolved by SDS-PAGE using a Criterion 4–15% Tris-HCl gradient gel (Bio-Rad, Hercules, CA). Thirty ZP, which were not incubated with the PAD ligand, were solubilized in 1X reducing SDS-PAGE loading buffer and served as a positive control. Samples were electro blotted onto a polyvinylidene difluoride membrane (cat. no. IPVH00010, EMD Millipore, Bedford, MA) and the membrane was blocked for 1 h at RT with shaking in 3% nonfat dry milk in TBST and then incubated overnight with shaking at 4°C with either 0.5 μg/ml rat anti-mouse ZP1 (M1.4, sc-32751, Santa Cruz Biotechnology, Santa Cruz, CA), 0.25 μg/ml rat anti-mouse ZP2 (IE-3, sc-32752, Santa Cruz Biotechnology), or 0.5 μg/ml rabbit anti-human ZP3 (H-300, sc-25802, Santa Cruz Biotechnology) in 3% nonfat dry milk in TBST. The membranes were washed 3 x 5 min in TBST with shaking, and incubated for 2 h at RT with either 1:40,000 goat anti-rat conjugated HRP (cat. no. 31470, ThermoScientific, Waltham, MA) or 1:40,000 goat anti-rabbit conjugated HRP (cat.no. 65–6120, Invitrogen/ThermoScientific, Waltham, MA) secondary antibodies diluted in 3% nonfat dry milk in TBST. Membranes were then washed 8 x 5 min in TBST and bound enzyme was detected by chemiluminescence (Supersignal West Pico, cat. no 34080, ThermoScientific, Waltham, MA).

### Negative Stain Electron Microscopy

Because intact ZP were too large and dense for visualization by negative stain electron microscopy, ZP were solubilized as previously reported prior to placing on grids [4]. Briefly, 3–5 ZP were transferred in a small volume (1–2 μl) into a 500 μl microfuge tube containing 5 μl 0.4 μg/ml chymotrypsin (C-4129, Sigma Chemical Co., St. Louis, MO) in 10 mM ammonium acetate and 0.5 mM CaCl_2_ pH 7.3. Samples were incubated for 1 h at 37°C in a humidified chamber. Samples were then dried onto 200 mesh carbon-coated copper grids (cat. no. 01810, Ted Pella, Redding, CA). Chymotrypsin (0.4 μg/ml) in 10 mM ammonium acetate/0.5 mM CaCl_2_ was also dried on a grid to serve as a control. Aβ1–40 aggregates were prepared by incubating 77 μM Aβ (cat. no. A-1156-1, rPeptide, Bogart, GA) in PBS in a shaking water bath at 37°C for 90 min. The resulting aggregate was sonicated on ice for 15 s, 60% duty cycle, power 2 with a Ultrasonics W-375 sonicator to break large aggregates into smaller fragments. Five μl of the Aβ solution diluted to 10 μg/ml was then pipetted onto a grid, and then wicked off after 5 min. All grids were rinsed 1 min with water, stained with 2% uranyl acetate (cat. no. 19481, Ted Pella, Redding, CA) for 1 min, and then rinsed with water again for 1 min before drying. Grids were examined with a Hitachi H-8100 transmission electron microscope (Hitachi, Dallas, TX).

### Powder X-ray Diffraction

Approximately 80 ZP were precipitated in 150 μl acetone overnight at -20°C. The sample was centrifuged at 17,200 g for 15 min at 4°C, the acetone discarded, and pellet resuspended in 150 μl cold acetone. The sample was centrifuged again, acetone discarded, and the pellet dried by SpeedVac (Savant, Farmingdale, NY) for 1 min. The pellet was resuspended in 10 μl 5mM ammonium acetate pH 7.3, and pulled into a 0.7 mm quartz capillary tube. The sample was then allowed to air dry in the presence of desiccant. At 5 mM ammonium acetate is a volatile buffer and does not form crystals that would interfere with the diffraction pattern. X-ray diffraction data were acquired using a Rigaku Screen Machine (Rigaku, The Woodlands, TX) X-ray generator utilizing CuKa radiation (1.5418 Å) with a focusing mirror (50kV, 0.6mA) and mercury CCD detector. The distance from sample to detector was 75mm.

### AmylPred2

Primary sequences of mouse ZP1 (UniProt Q62005), ZP2 (UniProt P20239), and ZP3 (UniProt P10761) were analyzed by the AmylPred2, an algorithm for consensus prediction of amyloidogenic determinants in polypeptide sequences (http://aias.biol.uoa.gr/AMYLPRED2) [23]. Briefly, AmylPred 2 uses the consensus, defined as the hit overlap of 5 of 11, different methods that are known or specifically designed to predict features related to the formation of amyloid fibrils. Included in the 11 methods are several established web based tools for predicting amyloid including AGGRESCAN [24], Pafig [25], TANGO [26], and Waltz [27]. AmylPred2 was also used to predict amyloidogenic regions in the ZP polymerization domains of homologs of mouse ZP3 including *Haliotis rufescens* (red abalone) vitelline envelope receptor of lysin (VERL repeat 10 and repeat 23) (UniProt Q8WR62), *Oncorhynchus mykiss* (rainbow trout) vitelline envelope protein gamma (Genbank AAF71260), *Xenopus laevis* gp41 (UniProt Q91728), *Coturnix japonica* (Japanese quail) glycoprotein C (GenBank BAA25637), and human ZP3 (UniProt P21754). The ZP-N domain in *Saccharomyces cerevisiae* α-agglutinin/Sag 1 (UniProt P20840) was also examined.

## Results

**Fig 1:**
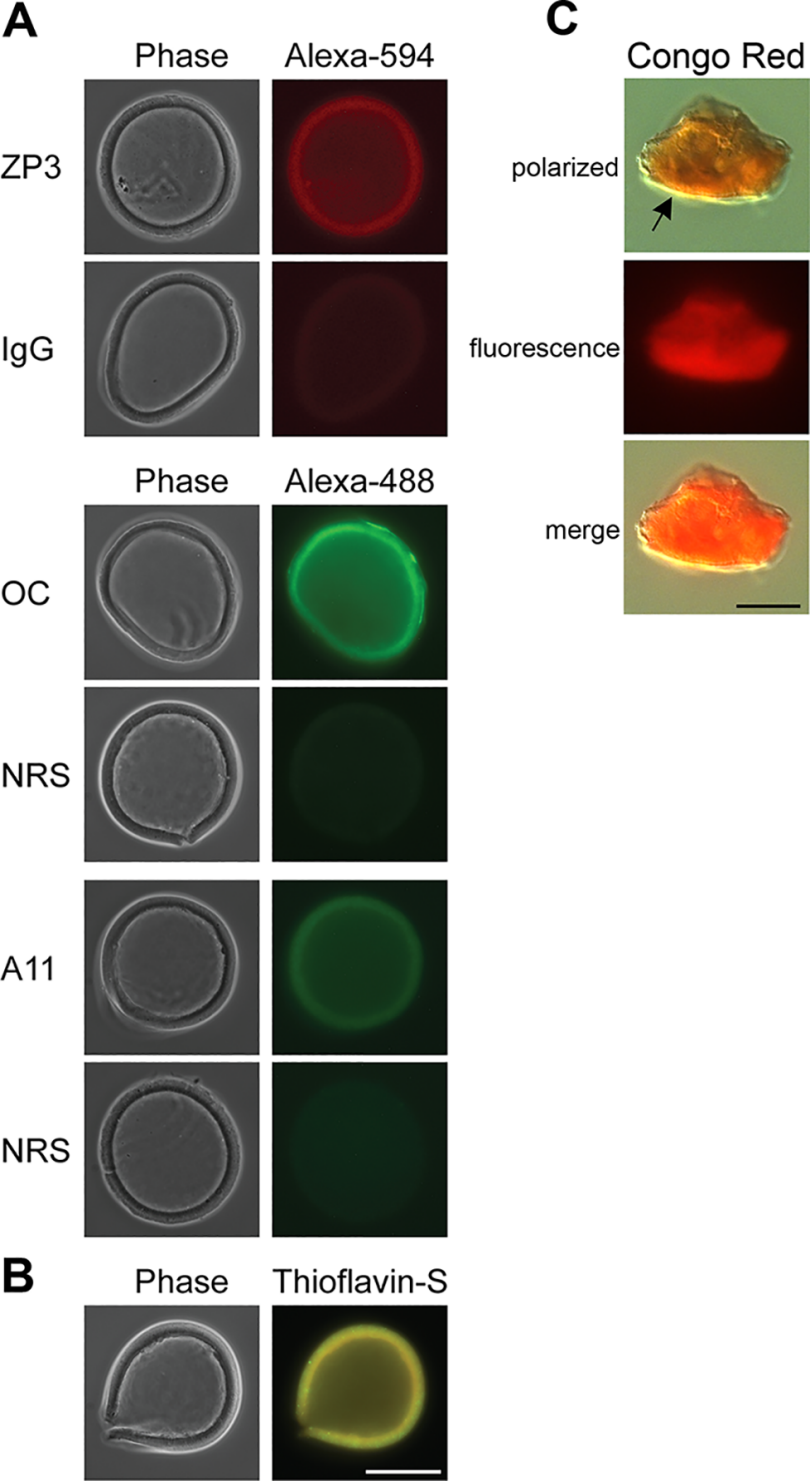
Amyloidogenic properties of the mouse ZP. A) The detection of amyloids in isolated mouse ZP was carried out using the amyloid conformation-dependent antibodies anti-fibrillar OC and anti-oligomer A11 in immunofluorescence analysis. Normal rabbit serum (NRS) served as a control. The anti-ZP3 antibody was used as a marker for the ZP with normal goat IgG serving as a control antibody. The corresponding phase images are shown for each fluorescent image. B) Intact ZP stained with 0.1% thioflavin S to detect amyloids. Scale bar = 50 μm. C) ZP pellets stained with 0.2% Congo Red showed yellow-green birefringence (arrow) when examined under polarizing light and bright red fluorescence when examined with UV light. Scale bar = 10 μm.

ZP were isolated from oocytes and examined by immunofluorescence using the conformation-dependent antibodies anti-amyloid oligomers A11, which recognizes early, immature forms of amyloid including oligomers, and anti-amyloid fibrils OC, which recognizes mature forms of amyloid including fibrils, to determine if amyloid was present in the ZP [28]. OC and A11 positive immunofluorescence, not present with the control normal rabbit serum (NRS), was detected in the isolated ZP suggesting the presence of both mature and immature forms of amyloid (Fig 1A). Immunofluorescence analysis with an anti-ZP3 antibody confirmed the isolated structure was the ZP (Fig 1A). Normal goat immunoglobulin G (IgG) did not reveal any staining demonstrating the specificity of the ZP3 antibody. Isolated ZP were also stained with thioflavin S, a conformation-dependent dye that exhibits enhanced fluorescence when bound to cross β sheet structures typical of amyloid [29]. Bright thioflavin S staining was detected in the ZP supporting our immunofluorescence observations that the ZP is likely an amyloid (Fig 1B). Also, isolated/dispersed ZP pellets stained with the conformation-dependent dye Congo Red exhibited the characteristic yellow-green birefringence when examined under polarizing light indicating the presence of a cross β-sheet structure typical of amyloid (Fig 1C) [30].

### Detection of ZP glycoproteins in the ZP amyloid

**Fig 2:**
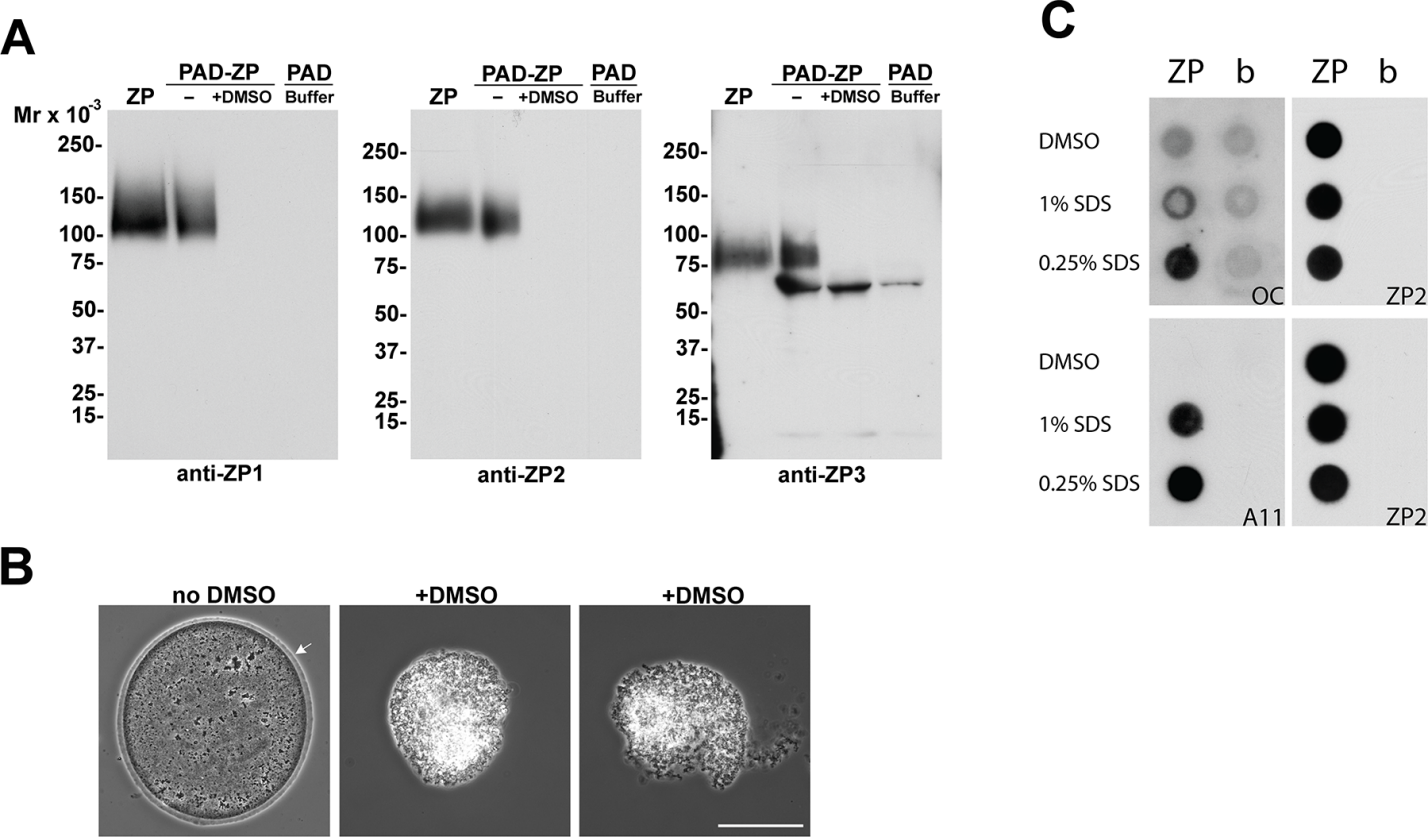
Identification of proteins in the ZP amyloid. A) Thirty isolated ZP were treated with (+) or without (-) DMSO followed by incubation with the PAD ligand and eluted proteins examined by immunoblot using anti-ZP1, ZP2, and ZP3 antibodies. ZP, an equivalent number of ZP placed directly in SDS-PAGE loading buffer without PAD incubation. Buffer, PBS only incubated with PAD beads. Molecular weight markers indicate kDa. B) phase images of oocytes incubated in the presence (+) or absence (no DMSO) of 90% DMSO for 2 minutes at room temperature. Arrow indicates ZP. Scale bar = 50 μm. C) Equal numbers of ZP were exposed to 90% DMSO, 1% SDS or 0.25% SDS prior to spotting on nitrocellulose using a dot blot apparatus. Membranes were then incubated with the amyloid anti-fibrillar OC and anti-oligomer A11 antibodies. Dot blots were rehybridized with anti-ZP2 antibody to confirm the presence of ZP protein in each well. b, buffer.

To determine if the individual ZP proteins in the ZP are amyloid, pulldown experiments were performed in which isolated ZP were incubated with the protein aggregation disease (PAD) ligand, which interacts with aggregated proteins via repeating charged and hydrophobic groups and specifically binds amyloids including fibrillar and oligomeric forms but not natively folded or monomeric proteins [31]. As shown in Fig 2A, immunoblot demonstrated that all three ZP proteins bound to the PAD ligand suggesting they are amyloid. Immunoblot analysis was also carried out on an equal number of ZP that were not incubated with the PAD ligand. Because the intensity of the chemiluminescent signal for each protein in the nonPAD bound ZP was the same as that in ZP that were incubated with the PAD ligand, suggested that the majority, if not all of each ZP protein, is in an amyloid structure in the ZP with little to no monomeric forms present. Although PAD pulldown experiments were carried out in the presence of denaturants including guanidine-Cl, which is a component of one of the PAD reagents, we cannot rule out if the ZP proteins bound to the PAD ligand are in complexes. A 60 kDa protein detected with the ZP3 antibody in PBS buffer only incubated with PAD ligand likely reflected crossreactivity with albumin present in one of the PAD reagents.

To further confirm the ZP proteins are amyloid, we repeated the PAD pulldown experiments after first treating the isolated ZP with 90% DMSO. DMSO has been used in studies of amyloids to break hydrogen bonds and disrupt aggregated beta sheets [32]. Treatment of ZP with DMSO abolished the binding of all three ZP proteins to the PAD ligand suggesting that DMSO reversed the ZP amyloid to preoligomers or monomers that were incapable of binding (Fig 2A). Using the known amyloidogenic proteins CRES and cystatin C, we have previously determined that DMSO does not alter the binding specificity of the PAD ligand such that monomeric or natively folded proteins can bind (Whelly and Cornwall, unpublished observations). In support that ZP structure is affected by DMSO, the ZP of intact oocytes exposed to 90% DMSO rapidly dissolved within 2 minutes leaving a fragile contracted oocyte that was easily broken apart (Fig 2B). To extend our studies of ZP amyloid stability, isolated ZP were exposed to different concentrations of SDS or DMSO and then examined for amyloids with OC and A11 antibodies in dot blot analysis which allowed us to capture all forms of amyloid including those that might be released during exposure to denaturants. ZP mildly solubilized by 0.25% SDS showed strong OC and A11 immunoreactivity similar to that observed by immunofluorescence analysis in isolated ZP (Fig 2B). In contrast, ZP exposed to 1% SDS resulted in a decrease in OC immunoreactivity, suggesting the loss of mature forms of amyloid. However, immature forms of amyloid as indicated by A11 immunoreactivity remained, showing that the ZP amyloid was somewhat resistant to SDS. Similar to that observed in the PAD pulldown experiments, exposure of ZP to DMSO resulted in the loss of both OC and A11 immunoreactivity suggesting that DMSO reversed the ZP amyloid structure such that both immature and mature forms of amyloid were no longer present (Fig 2B). Rehybridization of the dot blot with ZP2 antibody confirmed the presence of ZP protein in each spot on the membrane.

### Analysis of ZP structure

**Fig 3:**
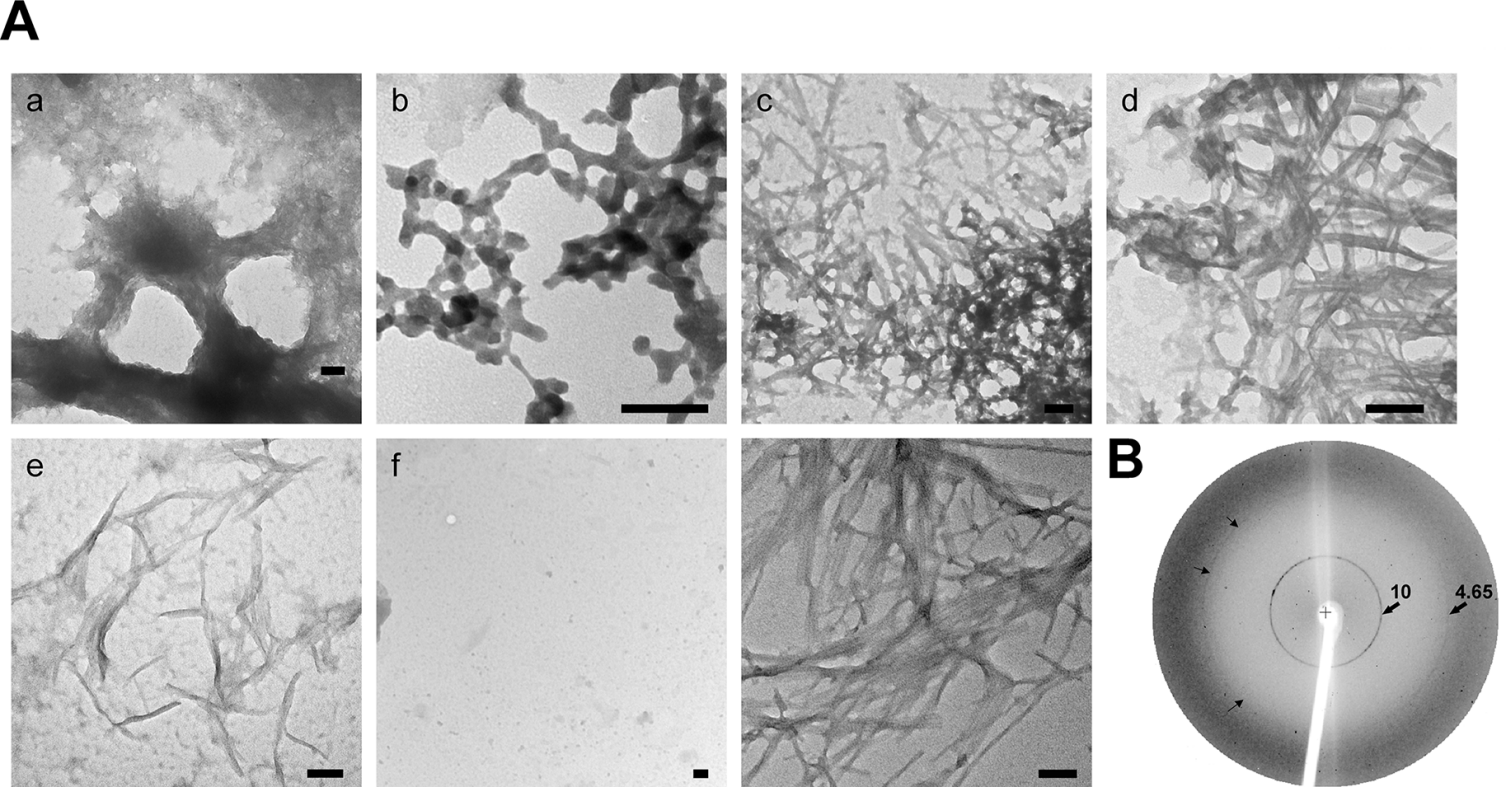
ZP exhibit structural characteristics of amyloid. Aa-Ae) Isolated ZP were digested with chymotrypsin followed by spotting on to grids and staining with 2% uranyl acetate. TEM micrographs show the various amyloid-like structures observed in the dispersed ZP. Af) Negative control in which buffer containing chymotrypsin but not ZP was spotted on to a grid. Ag) TEM micrograph of human Aβ amyloid fibrils. Scale Bar = 100 nm. B) X-ray diffraction of mouse ZP. Numbers represent Angstroms. Small arrows indicate the 4.65Å reflection.

To examine the structure of the ZP, isolated ZP were treated with chymotrypsin, which previously has been shown to expose ZP filaments [4], and then examined by negative stain electron microscopy. After 1 hour incubation, we observed the ZP in various states of disaggregation including the presence of large dense branched structures that appeared to be unwinding into a loose matrix (Fig 3Aa), branched polygon/hexagon structures that gave the appearance of beads on a string (Fig 3Ab), dense meshworks of packed fibrils (Fig 3Ac-d), and small clusters of fibrils (Fig 3Ae), characteristic of amyloid. The fibrils present in the dispersed ZP were similar to the clusters of individual and stacked fibrils observed in a sample of human Aβ amyloid fibrils prepared for comparison (Fig 3Ag). The diameter of the individual ZP fibrils in Fig 3Ae were 10–18 nm in diameter, similar to what has been previously described for amyloid fibrils and which was comparable with the diameter of the Aβ amyloid fibrils in Fig 3Ag [17]. Chymotrypsin spotted on to the grid in the absence of ZP did not show any fibrils (Fig 3Af). Because chymotrypsin was shown to preferentially digest ZP1 in isolated ZP, the amyloid fibril morphology we observed by negative stain EM may have been affected [4].

Isolated ZP were also examined by powder X-ray diffraction which revealed reflections at both 10 Å and 4.6Å, characteristic of a cross-β pattern typical of amyloids (Fig 3B) [33]. The 4.7Å reflection represents spacing between strands in a β-sheet while the 10Å reflection represents spacing between β- sheets. The diffuse broad ring observed at resolution lower than 4.65Å has been observed in diffraction patterns of other amyloids isolated from biological samples and was attributed to a small amount of lipid remaining from the isolation procedure [19]. Together, the structural studies suggest the presence of amyloid in the mouse ZP.

### Identification of amyloidogenic sites in the ZP proteins

**Fig 4:**
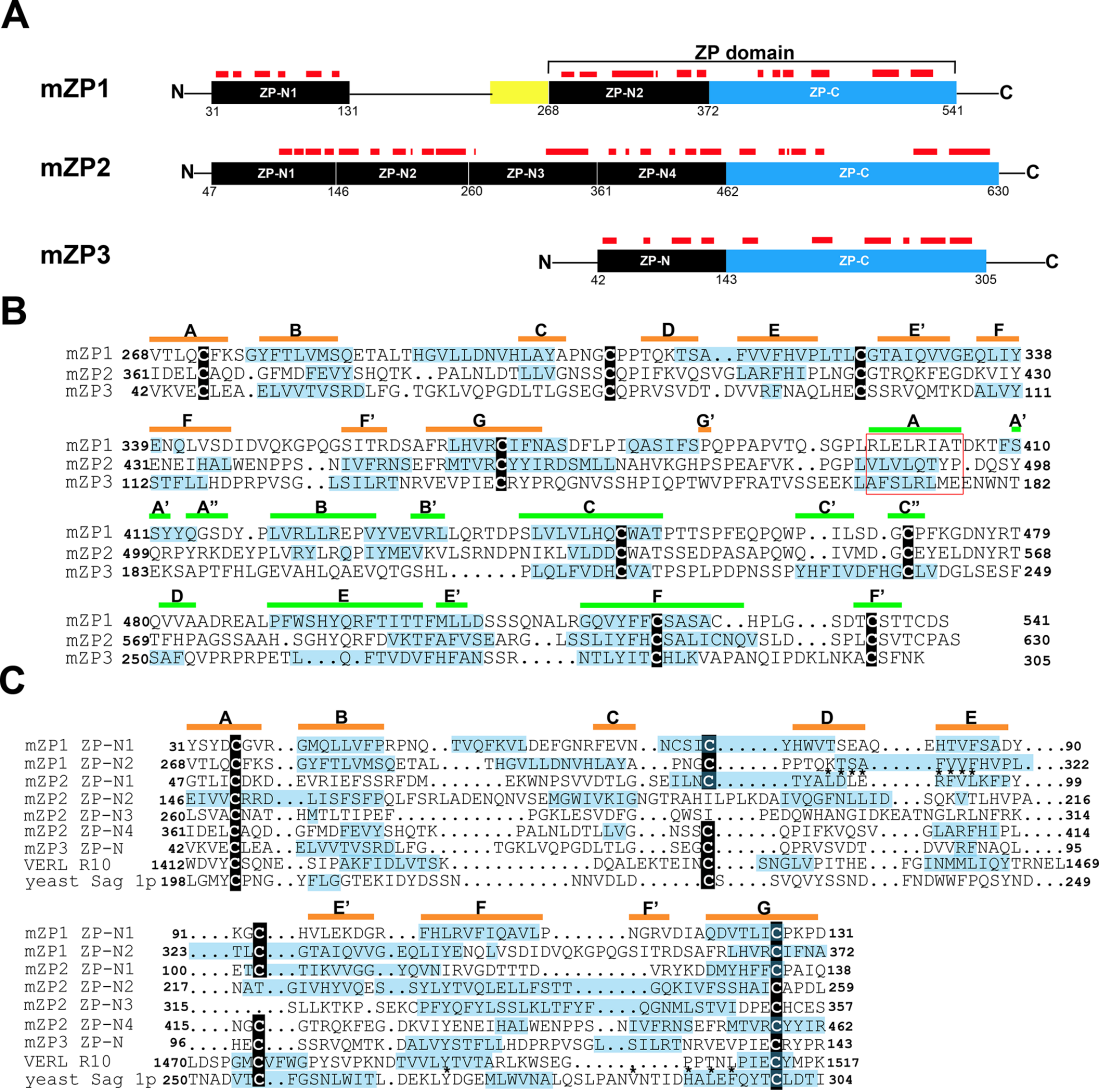
Identification of amyloidogenic regions in mouse ZP proteins. A) Schematic diagram of mature ZP1, ZP2, and ZP3 with amyloidogenic regions predicted by AmylPred 2 indicated as red bars above the individual domains. Yellow box, trefoil domain. Numbers signify amino acid number. B) Structure based sequence alignment of the ZP polymerization domain of mouse ZP1 (aa 268–541), ZP2 (aa 361–630), and ZP3 (aa 42–305) showing amyloidogenic sites (blue highlighting), as predicted by the Amylpred2 algorithm. Cysteine residues are noted by black boxes. The internal hydrophobic patch (IHP) is indicated by a red box [35]. The β-strand secondary structure based on the crystal structure of chicken ZP3 is noted by orange bars (ZP-N subdomain) and green bars (ZP-C subdomain) above the amino acid sequences [8]. C) Structure based alignment of ZP-N domains in mouse ZP1 (N1, N2), ZP2 (N1-N4), ZP3 (N), abalone VERL repeat 10 (R10) and yeast α-agglutinin/Sag 1p showing predicted amyloidogenic sites in blue highlighting as determined by the AmylPred2 algorithm. The β-strand secondary structure, based on structure of mZP-N is indicated by orange bars above the amino acid sequence [7]. Cysteine residues are noted by black boxes. *, indicate sites essential or important for ZP2-sperm and α-agglutinin-a-agglutinin binding [7, 36].

We next analyzed the primary sequences of mouse ZP1, ZP2 and ZP3 using the algorithm Amylpred2 to identify potential amyloidogenic sites throughout each protein. Amylpred2 uses the consensus of 11 different methods that have been developed to predict features relating to amyloid fibrils including amyloidogenic sequence pattern, beta- strand contiguity, average packing density, and includes the amyloid prediction programs AGGRESCAN, TANGO, and WALTZ [23]. Amyloidogenic segments are usually short peptide sequences of 4–12 amino acids that alone can form fibrils as well as drive fibril formation in their parent proteins [34]. The type of the amino acid in context with its neighbors contributes to the amyloidogenic properties of the segment allowing very different protein sequences to adopt similar tertiary structures. Fig 4A shows a schematic diagram of each mature ZP protein including the ZP polymerization domain (ZP-N + ZP-C) and N-terminal ZP-N repeat domains in ZP1 and ZP2 with regions predicted to be amyloidogenic indicated as red bars above each domain.

Several regions within the ZP polymerization domains of ZP1, ZP2, and ZP3 were predicted to form amyloid (Fig 4A). Using the crystal structure of the chicken ZP3 polymerization domain as a model, a structure-based alignment of the ZP polymerization domains from ZP1, ZP2, and ZP3, showed, despite low amino acid sequence similarity, a striking conservation of amyloidogenic sites, as indicated by blue highlighting, in both the N-terminal and C-terminal ZP subdomains suggesting possible conserved functions between the ZP proteins (Fig 4B). Other amyloidogenic regions that were distinct to each protein, or common to just two of the three proteins, were also detected suggesting subunit-specific functions (Fig 4B). Most of the amyloidogenic sites mapped to amino acid stretches predicted to form β-strands based on ZP3 crystal structure including regions important for interactions between ZP subdomains. The ZP3 polymerization domain crystallized as a homodimer with the dimer interphase shown to be an antiparallel β-sheet formed by the ZP-N F’ β-strand of one molecule and the ZP-C E’ β-strand of another [8]. In the ZP2 and ZP3 polymerization domains, the amino acids that compose both the ZP-N F’ strand and the ZP-C E’ strand were predicted to be amyloidogenic suggesting subunit interactions could be mediated by the amyloidogenic properties of these regions (Fig 4B). In ZP1, however, the ZP-N F’ strand was not predicted to be amyloidogenic suggesting other sites may be important for its fibril assembly. In addition, a conserved seven amino acid patch containing hydrophobic residues, previously described by Jovine et al., 2004 [35] as the internal hydrophobic patch (IHP), and shown to be essential for mouse ZP3 assembly, was predicted to be amyloidogenic in ZP2 and ZP3 but not in ZP1 (Fig 4B, red box). The IHP is also at the ZP3 dimer interphase and comprises the A β-strand of the ZP-C domain [8].

In ZP1 and ZP2, regions proximal to the ZP polymerization domain also contained numerous sites that were predicted to form amyloid (Fig 4A). The N-terminal half of ZP2 consists of three ZP-N repeat domains arranged in tandem while the N-terminal half of ZP1 consists of one ZP-N repeat domain [37]. The ZP-N repeats have been shown to bind sperm and regulate gamete recognition [38–39]. The amyloidogenic regions were primarily localized to these ZP-N repeat domains suggesting the amyloid structure may play a role in gamete recognition (Fig 4A). Additional copies of ZP-N have also been found in the N-terminal region of egg coat homologs of ZP3, notably within the N-terminal regions of abalone vitelline envelope subunits VERL which also participate in sperm binding [40]. More recently, FUGUE threading analysis identified a VERL-like ZP-N repeat in the yeast mating protein α-agglutinin/Sag 1p, which functions by binding to a-agglutinin in haploid cells of opposite mating type [10]. Taken together, this suggests the ZP-N fold is a conserved 3D structure that mediates gamete recognition including parallel processes in yeast. It is important to note, however, that threading analyses merely estimates the possibility that a known 3D fold is adopted by a given sequence and does not directly determine whether the corresponding proteins share common ancestry or is the result of convergent evolution [10].

To examine more closely the potential role of the amyloid structure in ZP-N domain function, we used AmylPred 2 to identify amyloidogenic sites in the ZP-N domains from mouse ZP1, ZP2 and ZP3 as well as in abalone VERL ZP-N repeat 10 and the ZP-N like domain in yeast α-agglutinin/Sag 1p. As shown in Fig 4C, despite low sequence similarity, there was a conservation of sites throughout the ZP-N domain that were predicted to form amyloid across all species suggesting the ability to adopt an amyloid structure may be integral for ZP-N domain function. In particular, the crystal structure of the ZP-N domain of mouse ZP3 strongly predicts the E’-F-G extension to be involved in protein-protein interactions [7]. The presence of multiple amyloidogenic sites in the E’-F-G extension and their conservation in yeast, abalone, and mouse ZP-N domains suggests that the amyloid structure may participate in these interactions. Furthermore, previous studies have suggested that amino acids that are essential for yeast α-agglutinin to bind to a-agglutinin are located on the same face of the ZP-N fold as ZP2 residues involved in binding to sperm [10]. It is intriguing that in both mouse ZP2 ZP-N1 and α-agglutinin these binding sites, as indicated by asterisks in Fig 4C, map to sequences that are predicted to form amyloid suggesting a possible role for amyloid in gamete recognition and parallel processes in yeast.

### Evolutionary conservation of amyloidogenic propensity in egg coat/ZP protein polymerization domain

**Fig 5:**
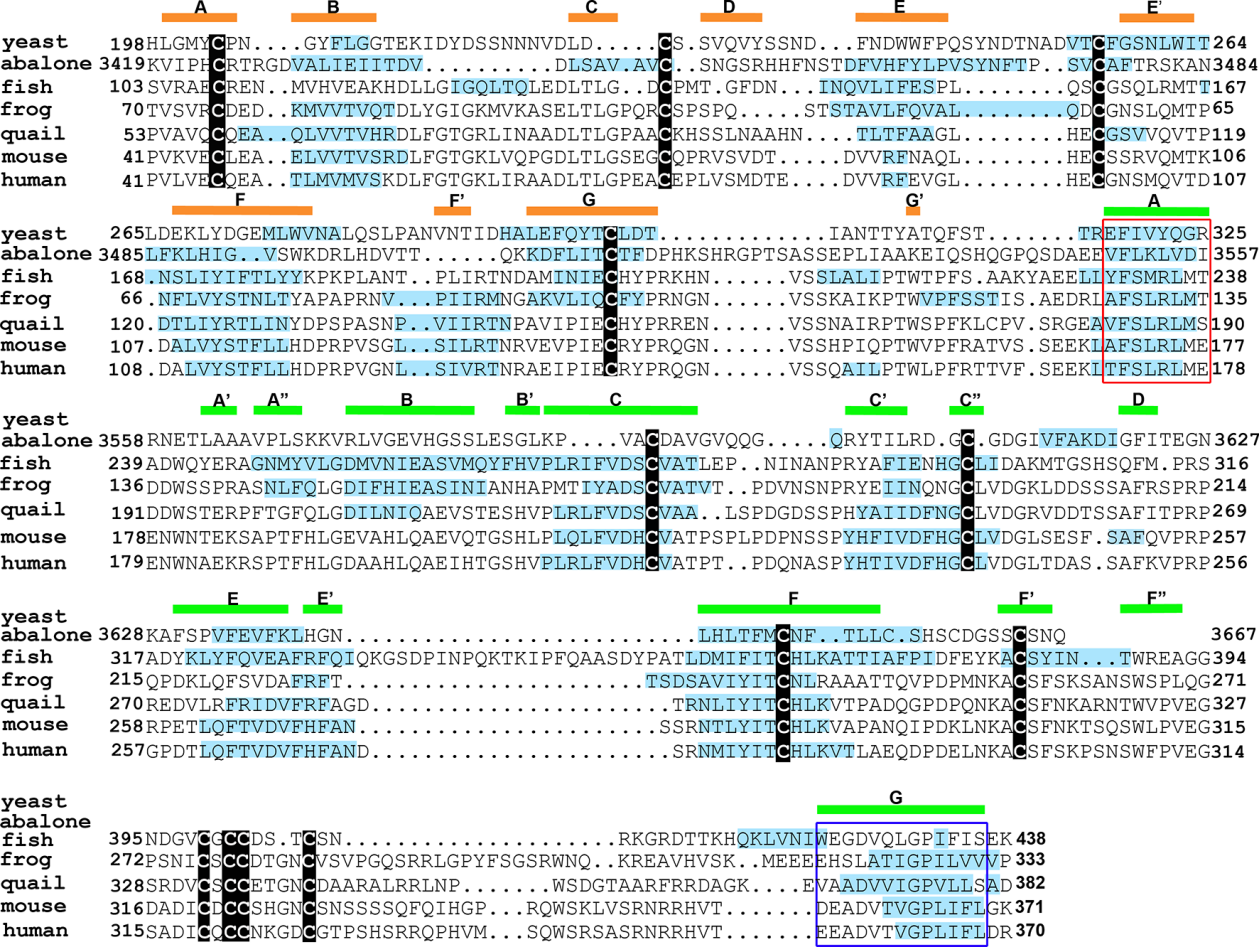
Evolutionary conservation of amyloidogenic sites in the ZP polymerization domain of ZP3 homologs. Structure based sequence alignment of the ZP polymerization domain of mouse ZP3 and ZP3/egg coat homologs from *Haliotis rufescens* (red abalone) vitelline envelope receptor of lysin (VERL repeat 23), *Oncorhynchus mykiss* (rainbow trout) vitelline envelope protein gamma, *Xenopus laevis* (frog) gp41, *Coturnix japonica* (Japanese quail) glycoprotein C and human ZP3 with the amyloidogenic sites as predicted by AmylPred2 indicated by blue highlighting. The ZP-N domain in *Saccharomyces cerevisiae* α-agglutinin/Sag 1 is also shown with its predicted amyloidogenic sites. Cysteine residues are noted by black boxes. The internal hydrophobic patch (IHP) is indicated by a red box and the external hydrophobic patch (EHP) is noted by the blue box [35]. The β-strand secondary structure based on the structure of chicken ZP3 is noted by orange bars (ZP-N subdomain) and green bars (ZP-C subdomain) [8].

A species comparison of egg coat/ZP proteins from abalone, fish, frog, quail, rat, and human demonstrated that they all shared a ZP polymerization domain [41]. Although the overall sequence homology was relatively low between the six taxa, they all shared eight cysteine residues found in ZP type I domain proteins such as mouse ZP3. Based on previous work suggesting that the egg coats surrounding fish and silk moth oocytes were functional amyloids [12–14], we used Amylpred2 to examine the ZP polymerization domain from ZP3 and the homologs of ZP3 in the taxa studied previously, with mouse replacing the rat, to determine if the propensity to form amyloid was conserved in egg coats/ZP between invertebrates and vertebrates. Furthermore, the ZP-N domain of yeast protein α-agglutinin, was also included for comparison [42]. As shown in Fig 5, throughout the ZP polymerization domain there was a notable conservation between all six taxa from abalone to human in the regions of the ZP domain that were predicted to form amyloid. In particular, this included a conservation of the amyloidogenic properties of the amino acids comprising the IHP, which is located at the ZP3 dimer interphase, and which was shown to be essential for mouse ZP3 fibril assembly [8, 35]. Although structural studies of chicken ZP3 revealed that the IHP comprises the A β-strand of the ZP-C domain [8], which is lacking in yeast, the presence of a region analogous to the IHP in yeast α-agglutinin and its conserved amyloidogenic properties suggests this stretch of amino acids may also be functionally important for α-agglutinin structure. The amyloidogenic properties of the amino acids forming the ZP-N F’β-strand and the ZP-C E’ β-strand, whose interactions formed the antiparallel β-sandwich of the chicken ZP3 homodimer, were also conserved across several species. However, although the amino acids forming the ZP-C E’ β-strand were predicted to form amyloid in all taxa except abalone, those composing the F’ β-strand were predicted to be amyloidogenic only from frog to human ZP3. Amino acids forming the external hydrophobic patch (EHP), which comprise the G β-strand of the ZP-C domain, also exhibited conserved amyloidogenic properties across species (Fig 5). Although the EHP does not participate in the formation of the ZP fibril, the EHP has been proposed to function as a mediator of ZP3 fibril assembly by binding to and inhibiting IHP interactions thereby preventing premature polymerization [35].

## Discussion

Our studies presented herein suggest the mouse ZP is a functional amyloid. Using approaches commonly used to identify amyloids, including conformation-dependent reagents, electron microscopy, and X-ray diffraction, our studies suggest that all three ZP proteins are amyloidogenic and contribute to the formation of the ZP amyloid. These results are consistent with previous reports indicating that all ZP protein can form fibrils and that ZP1-ZP3 and ZP2-ZP3 heterodimers contribute to the formation of the ZP matrix [11, 43]. Indeed, using an algorithm to predict regions that have the propensity to form amyloid, we identified numerous regions in all three ZP proteins that were aggregation prone including regions in the ZP polymerization domain which is essential for fibril assembly.

The ZP polymerization domain is a C-terminal region of approximately 260 amino acids that is present in ZP/egg coat proteins as well as many other proteins of diverse function outside of the reproductive tract [44]. Similar to the ZP/egg coat proteins, ZP domain proteins unrelated to fertilization form fibrils and extracellular matrices and the ZP domain has been implicated in this process [45–46]. Crystallization of the mouse ZP3 ZP-N subdomain and chicken ZP3 polymerization domain has provided considerable insight into ZP assembly [7–8]. The ZP-N and ZP-C subdomains are rich in β-strands and adopt an IgG-like protein fold with a common topology despite having no significant sequence similarity [8]. A prerequisite step in ZP3 biogenesis is the formation of a dimer involving contacts between the ZP-N and ZP-C subdomains from opposite molecules. Specifically, an antiparallel β-sheet is formed by the ZP-N F’- β strand of one ZP3 molecule and the ZP-C E’ β- strand from another [8]. Our studies showed that in both ZP2 and ZP3, the amino acids comprising these interacting β-strands have the propensity to form amyloid suggesting that amyloidogeneis may be the mechanism that drives this interaction and ultimately fibril assembly. Remarkably, similar to the ZP3 polymerization domain which crystallized as a domain-swapped dimer, many other amyloidogenic proteins also crystallized as domain swapped dimers including cystatin C, RNAse A, β_2_-microglobulin and the SH3 domain of c-src, leading to the suggestion that domain swapping may be an integral first step in amyloid assembly [47–49]. Thus, despite a lack of similarity in the amino acid sequences between the ZP-N and ZP-C subdomains, there is a significant conservation of sites that are predicted to be amyloidogenic, which would allow for the common topology between these two subdomains.

Detailed studies of mutant and truncated mouse ZP3 identified a conserved duplicated motif of seven amino acids called the internal hydrophobic patch (IHP), which comprises the A β-strand of the ZP-C domain, and which is vital for ZP3 assembly [35, 50]. The amino acids composing the IHP in ZP2 and ZP3, are predicted to be amyloidogenic suggesting that the propensity for these regions to form amyloid may also contribute to the assembly of ZP fibrils. The IHP in ZP1, however, is not predicted to be amyloidogenic suggesting that the regions that mediate ZP1 aggregation are distinct. In all three ZP proteins, in addition to the amyloidogenic sites mentioned, additional stretches of amino acids throughout the ZP-N and ZP-C subdomains of the ZP polymerization domain were predicted to be amyloidogenic. It is possible that multiple amyloidogenic sites dispersed throughout the ZP domain are required for fibril assembly. An analysis of amyloidogenic sites within known amyloid-forming proteins including Aβ, tau, and IAPP, showed that several proteins contained more than one fibril-forming segment. In vitro, these amyloidogenic segments adopted different amyloid structures (parallel β-sheets versus antiparallel β-sheets) despite being from the same protein [34]. Based on these results, it was proposed that fibrils formed from proteins with multiple amyloidogenic sites may possess more than one type of β-sheet structure resulting in elaborate sheet structures. Alternatively, protein fibrils may contain β-sheets generated from more than a single amyloidogenic segment or there may be polymorphic fibrils of the same protein [34]. The presence of multiple amyloidogenic sites in the ZP polymerization domain of each ZP protein may be necessary to direct heterodimer formation, specifically the formation of ZP1-ZP3 and ZP2-ZP3 amyloid fibrils, ultimately allowing the formation of a complex ZP matrix. It is also conceivable that other amyloidogenic sites may be important for postfertilization events allowing the ZP fibril assemblies to be reorganized and form the physical barrier to prevent polyspermy.

In addition to the ZP polymerization domain, our studies revealed that the ZP-N repeat domains also possess multiple stretches of amino acids that are predicted to form amyloid and that several of these sites are conserved across species. These sequences localize to defined β-strand structures some of which have proposed roles in protein-protein interactions. Indeed, studies both in mammals and other species suggest that the ZP-N repeat domains play important roles during gamete recognition [38–39]. Our studies showed that amino acid sequences implicated in ZP2 ZP-N binding to sperm as well as those critical for yeast α-agglutinin binding to a- agglutinin were predicted to be amyloidogenic suggesting a role for the amyloid structure in these cellular interactions.

A comparison of the ZP polymerization domains from ZP3 homologs from several species including marine invertebrates and humans showed that many of the amyloidogenic regions we identified in mouse ZP3 were conserved suggesting that the egg coat/ZP from these species are also amyloid which is consistent with their also being described as fibrillar matrices. These results are also consistent with previous reports describing the fish and silk moth egg coat as amyloids [12–14]. Although yeast do not possess an egg coat or ZP equivalent, haploid cells express complementary sexual agglutinin proteins on their surface that mediate cell-cell contact to promote fusion during mating [51]. Significantly, the ZP-N subdomain of the *Saccharomyces cerevisiae* mating protein, α-agglutinin, possessed several amyloidogenic regions that were similar to those present in egg coat/ZP proteins also suggesting a role for the amyloid structure in yeast mating. In particular, the ZP domains from all species, including yeast possessed a short amyloidogenic region that was analogous to the IHP described for mouse and human ZP3 [35]. Conservation of an IHP-like site, that in mouse is essential for ZP assembly, suggests that similar mechanisms may be used by other species to regulate their egg coat/ZP assembly, or in yeast, the assembly of its mating protein on the cell surface [10, 35]. That this site is also a highly conserved amyloidogenic region suggests that amyloid formation may be a conserved mechanism for assembly and function of egg coat/ZP/mating proteins.

Knowledge that many proteins unrelated to fertilization also contain ZP domains and are known to form fibrils and extracellular matrices would suggest that they too form amyloids for functional purposes. Many proteins that possess ZP domains are involved in cell-cell interactions and cell shape suggesting that amyloidogenesis may be a conserved mechanism for these basic cell biological processes [44, 52].

### Control of functional amyloid formation in the ZP

Other evidence supports our conclusion that the mouse ZP is an amyloid. In particular, the mechanisms that regulate ZP assembly are very similar to those that mediate the formation of other functional amyloids including those produced by bacteria. Although poorly understood, proteins that form functional or pathological amyloids are thought to follow similar aggregation pathways progressing from monomeric to soluble oligomeric to mature amyloid fibrils. Because the soluble oligomeric forms of many amyloidogenic proteins are cytotoxic, functional amyloids are thought to form under controlled conditions that prevent or minimize these intermediate forms. Studies of functional amyloids have revealed several distinct mechanisms to control amyloid formation including sequestration of the amyloid within an organelle, the requirement for interactions with other family members which act as nucleators initiating amyloid formation, and activation of amyloidogenesis via protein processing by proprotein convertases [16, 53]. All ZP proteins contain a C-terminal proprotein cleavage site and are processed by a proprotein convertase prior to their release into the extracellular space and fibril formation [2, 3]. An additional level of control may come from interactions between ZP proteins since, ZP3, containing a type I ZP domain, requires the presence of ZP1 or ZP2, containing type II ZP domains for polymerization [3, 54–55]. This is strikingly similar to the nucleation-dependent aggregation described for the bacterial curli family of amyloidogenic proteins in which the curli member CsgA requires the presence of the nucleator protein, CsgB to initiate fibril formation at the cell surface [56].

### Function of ZP amyloid during fertilization

The functional significance of the ZP as an amyloid may be several-fold. First, the inherent stability of amyloid and its resistance to denaturants and proteases would allow the ZP to survive despite being surrounded by proteases and hydrolases associated with sperm that are undergoing, or have undergone, the acrosome reaction. Indeed, following sperm binding, the ZP does not dissolve but rather sperm penetrate the ZP via a small slit that occurs in the matrix [57]. Because the ZP plays critical roles during early development/embryogenesis, its amyloid structure would ensure it remains relatively intact during sperm penetration enabling its later functions [58]. The ZP as an amyloid may also be significant from the standpoint of how sperm-ZP interactions occur. Microcrystals of amyloids show them to be composed of interlocking complementary structures known as steric zippers [34, 59]. Although microcrystals represent small peptides of amyloidogenic proteins, structural, nucleation, and mutational studies suggest they are representative of the amyloid structures formed from their parent proteins [34]. Given our previous observation that the mouse sperm acrosomal matrix, a structure that participates in ZP binding, is an amyloid [22], raises the intriguing possibility that sperm-ZP binding may be mediated by amyloid-amyloid interactions, possibly by a steric zipper mechanism. While studies are ongoing to address this, several cell adhesion molecules, many of which possess IgG protein domains, have been shown to establish cell-cell contacts by the formation of zippers [60].
